# Benign Multicystic Peritoneal Mesothelioma Presents as a Tumor in an Elderly Man: An Uncommon Diagnosis

**DOI:** 10.5339/qmj.2022.5

**Published:** 2022-02-22

**Authors:** Ahmet Askar, Ergin Erginoz, Ayse Hanım Yavuz, Vedat Durgun

**Affiliations:** ^1^Department of General Surgery, Istanbul University Cerrahpasa–Cerrahpasa School of Medicine, Istanbul, Turkey E-mail: achkar.dr@yahoo.com; ^2^Department of Pathology, Istanbul University Cerrahpasa–Cerrahpasa School of Medicine, Istanbul, Turkey

**Keywords:** Benign Multicystic Peritoneal Mesothelioma, Peritoneal Inclusion Cyst, Peritoneum

## Abstract

Background: Mesotheliomas are benign masses that can arise from any body parts that contain mesothelium, such as the abdominal, pelvic, pleural, and pericardial cavities. Benign multicystic peritoneal mesothelioma is a cystic tumor that arises from peritoneal mesothelial cells. It is a rare pathological entity, as only fewer than 200 cases have been reported. Benign multicystic peritoneal mesothelioma mainly occurs in women, and it is extremely rare in men. Its diagnosis and management are often challenging.

Case presentation: This report demonstrates a case of a 61-year-old man who presented to the outpatient clinic with persistent abdominal discomfort that progressed over the years. He had visited different clinics and was referred to a gastroenterologist because of a misdiagnosis. After an extensive clinical evaluation, we failed to provide a definitive diagnosis; thus, diagnostic laparotomy for possible intra-abdominal malignancy was performed. After successful surgical resection of the lesions, the pathology was found compatible with benign multicystic peritoneal mesothelioma.

Conclusion: Given its high recurrence rates and potential malignant transformation, meticulous and detailed surgical excision of the cystic lesions is of utmost importance to avoid repeated surgeries. Long-term follow-up is recommended.

## Introduction

Benign multicystic peritoneal mesothelioma (BMPM) is a rare disease, and it is extremely rare in men that can easily be overlooked by physicians. Neoplasia, chronic inflammation, irritation, and hormonal activity hypotheses have been suggested in the etiology, but the exact etiological factor has not been identified until today.^1^ BMPMs are usually asymptomatic, but with increased size, they can cause abdominal pain, discomfort, bloating, constipation, incontinence, and anorexia.^2^ Differential diagnoses include cystic lymphangioma (cystic hygroma), cystic endosalpingiosis, endometriosis, cystic adenomatoid tumors, and malignant peritoneal mesothelioma.^3,4^ Several treatment methods have been described in the literature demonstrating different clinical and long-term results. The treatment of choice is surgical excision.^5,6^ This report describes the case of a middle-aged man who presented with BMPM as a rare diagnosis.

## Case Report

This case report was approved by the institutional ethics committee.

**Figure 1.**
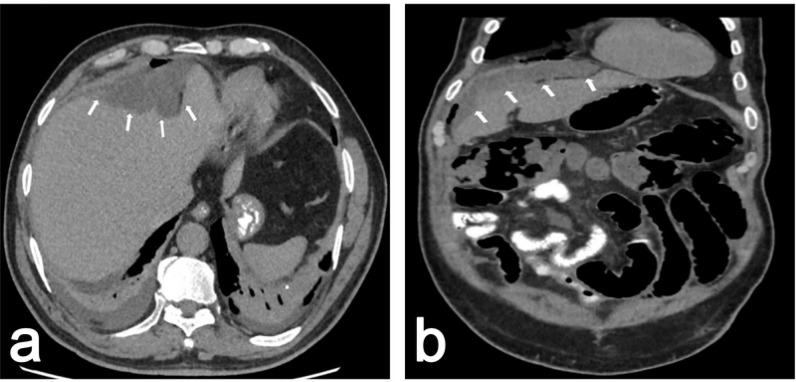
a: Axial computed tomography (CT) scan view of the abdomen, with the cystic mass compressing the liver and no pathologic contrast enhancement. The arrows show the margins of the tumor. **b:** Coronal CT scan view of the abdomen, with the cystic mass (4.7 × 11 × 10 cm) located at the subdiaphragmatic area adjacent to the liver, with no pathologic contrast enhancement. The arrows show the margins of the tumor.

**Figure 2.**
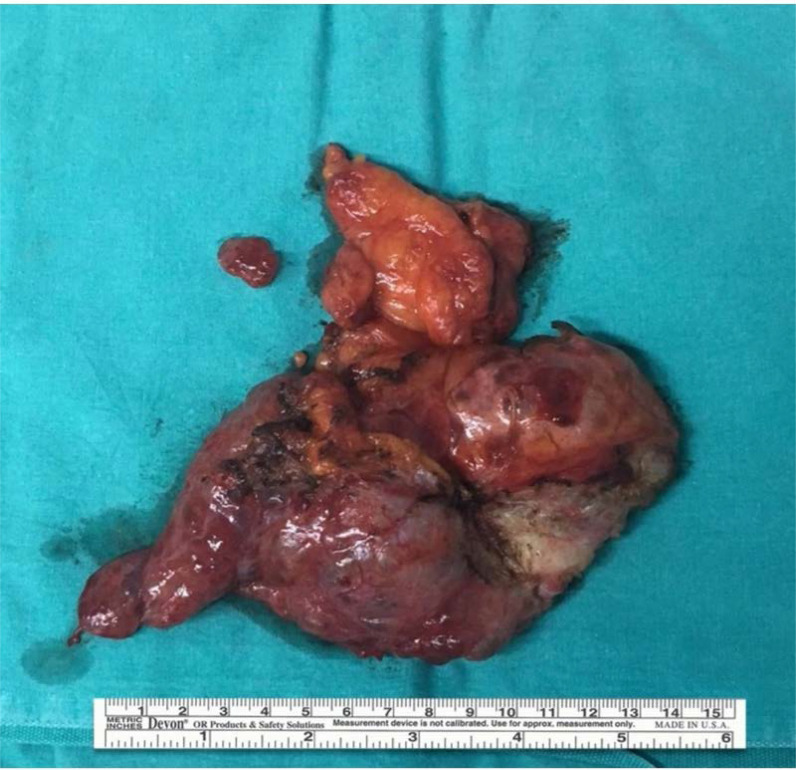
Gross appearance of surgically excised septated cystic tumor measuring 10 × 15 cm.

A 61-year-old male patient, who does not have a history of chronic disease, had consulted different outpatient clinics several times because of longstanding abdominal pain, discomfort, bloating, and loss of appetite. The patient was confined and given various medications for bloating and abdominal discomfort, which were prescribed by general practitioners but failed to relieve the patient's symptoms. In the history assessment, the patient stated that a mild pain persisted for several years, during which he was not admitted to any clinic. With the increase in the frequency of his symptoms in the last few weeks, he sought treatment from different outpatient clinics. He did not have a history of smoking, alcohol consumption, or previous abdominal surgery. The physical examination yields no remarkable findings, except for mild epigastric tenderness. Hemogram and biochemistry panel were within normal limits. Abdominal computed tomography (CT) detected multiple loculated collection areas in the abdomen, the largest of which was observed in the subdiaphragmatic area at the anterior of the liver, adjacent to hepatic segments 8, 4, and 5, and it was 4.7 × 11 × 10 cm in diameter. The mass did not contain any solid components or pathological contrast enhancement (Figure 1 a, b). The result of the indirect hemagglutination test was < 1/80 (negative < 1/160). With all these findings, we performed diagnostic laparotomy. During the operation, a yellowish-pink, soft, fluid-filled multiloculated cystic mass measuring 10 ×  15 cm adhered to the anterior surface of the liver, and numerous similar small cystic lesions were scattered on the right paracolic area and peritoneal surfaces. All cystic lesions were excised with LigaSure and electrocautery (Figure 2). For possible appendiceal malignancy, an appendectomy was performed.

**Figure 3.**
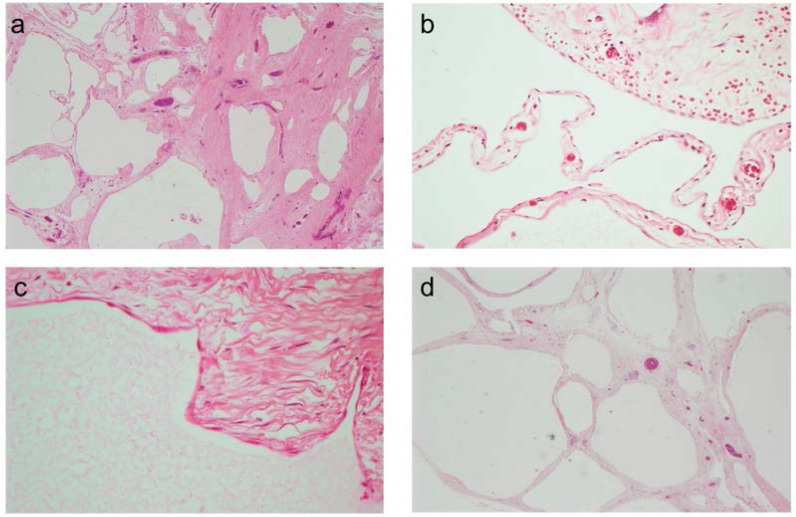
Microscopic examination using hematoxylin and eosin staining**. a:** Cysts separated by collagenous stromal septa. **b:** The cyst is lined by a single layer of bland, flat cells. **c:** The cyst is lined by a single layer of bland, flat cells. **d:** Numerous cysts were present in the peritoneum.

After an uneventful follow-up, the patient was discharged on postoperative day 5. Pathological examination of the excised specimen with hematoxylin and eosin demonstrated multiloculated, honeycomb-shaped, regular-walled cystic structures filled with clear or yellow serous fluid (Figure 3 a–d). Immunohistochemical stains or markers were not used. The appendix had a normal structure, and only lymphoid follicular hyperplasia was noted. Since all these findings were compatible with benign cystic lesions, the pathological diagnosis was BMPM. Ultimately, the patient was followed up in the outpatient clinic.

## Discussion

BMPM is a rare tumor caused by mesothelial proliferation in the serous surfaces covering the abdomen and pelvis.^1–3,6^ Approximately 200 cases have been reported in the medical literature since it was first described by Mennemeyer and Smith in 1979.^1,3,6^ BMPM is usually seen in childbearing young women, and it is rarely seen in men.^2,3,6^ Unfortunately, information about the etiopathogenesis of BMPM is limited; thus, some researchers consider them as neoplastic lesions, while others consider them lesions caused by inflammatory conditions such as the familial Mediterranean fever, peritoneal tuberculosis, and pelvic inflammatory disease.^1,3,5,6^ According to the inflammatory theory, chronic irritation after surgery or chronic inflammation suggests that cystic structures are formed following mesothelial cell entrapment and reactive proliferation. Another theory is the hormonal hypothesis that attributes the condition to sex hormone sensitivity because of the higher prevalence of BMPM in childbearing-age women, especially with those who have a history of endometriosis.^1,5,6^ BMPMs are usually asymptomatic; when enlarged, they can cause abdominal pain, discomfort, bloating, constipation, incontinence, and anorexia.^1,4^ Diagnosis is usually made by ultrasonography, abdominal CT, and abdominal magnetic resonance imaging.^1,3,4^ Even though the treatment of choice is surgical excision, there has been an ongoing debate regarding which treatment is more sufficient for BMPMs. While some favor surgery alone like the procedure presented herein, others prefer cytoreductive surgery, followed by hyperthermic intraperitoneal chemotherapy. Image-guided aspiration/sclerotherapy and medical treatment, such as oral contraceptives and gonadotropins are alternative modalities to surgery that can be useful in some cases.^1,3,5^ BMPM recurrence frequently occurs after treatment and requires repeated surgical interventions. Malignant transformation of BMPM is very rare, and only two cases have been reported to date.^2,3,5,6^

## Conclusion

Herein, we described a case with a rare diagnosis in a patient who had a misdiagnosis and thus went through inappropriate treatments repeatedly for a long period. This case indicates that BMPMs as rare benign tumors, with challenging diagnosis and management. Therefore, elderly patients with persistent abdominal discomfort should be evaluated with imaging modalities such as abdominal ultrasonography or CT for possible intra-abdominal pathologies. Given their high recurrence rates and risk of malignant transformation, we think that the treatment of BMPMs by highly experienced medical centers covers an important dimension.

### Declarations

### Competing interest

The authors declare no competing interests.

### Ethics approval and consent to participate

This case study was approved by the institutional ethics committee.

### Consent for publications

Written and verbal informed consent was obtained from a legally authorized representative(s) for anonymized patient information to be published in this article.

